# Natural language processing for geriatric syndromes: a systematic review of methods, applications, and challenges

**DOI:** 10.1186/s12911-026-03417-0

**Published:** 2026-03-12

**Authors:** Fahrurrozi Rahman, Imane Guellil, Abul Hasan, Huayu Zhang, Matúš Falis, Arlene Casey, Honghan Wu, Bruce Guthrie, Beatrice Alex

**Affiliations:** 1 Advanced Care Research Centre, University of Edinburgh, Edinburgh, UK; 2 Institute of Health Informatics, University College London, London, UK; 3 LifeArc, London, UK; 4 Usher Institute, School of Population Health Sciences, University of Edinburgh, Edinburgh, UK; 5 School of Health and Wellbeing, University of Glasgow, Glasgow, UK; 6 Edinburgh Futures Institute, University of Edinburgh, Edinburgh, UK; 7 School of Literatures, Languages and Cultures, University of Edinburgh, Edinburgh, UK; 8 Department of Cancer and Genomics, University of Birmingham, Birmingham, UK; 9 Nuffield Department of Primary Care Health Sciences, University of Oxford, Oxford, UK; 10 Institute for Neuroscience and Cardiovascular Research, University of Edinburgh, Edinburgh, UK

**Keywords:** Natural language processing, Geriatric syndromes, Systematic review

## Abstract

**Background:**

Geriatric syndromes (GS) are complex conditions that affect older adults and often require multidisciplinary assessment. Natural language processing (NLP) has emerged as a promising tool for extracting relevant clinical information from unstructured text in electronic health records (EHRs). However, the application of NLP in detecting and monitoring GS remains an evolving area of research. This systematic review explores the role of NLP in the identification and analysis of GS, examining its applications, methodologies, and effectiveness. Furthermore, this review discusses the existing challenges, limitations, and future directions to advance NLP applications in the GS research.

**Methods:**

We conducted a systematic literature search across ten databases to identify studies that applied NLP to GS detection. Articles were screened using predefined inclusion and exclusion criteria, and relevant studies were evaluated for quality using PROBAST. Data were extracted on study characteristics, datasets, annotation processes, NLP approaches, performance metrics, population demographics, and clinical applications. A PRISMA flow diagram was used to illustrate the study selection process.

**Results:**

A total of 65 studies were included, where the majority of the studies used traditional rule-based and machine learning approaches. Publicly available datasets were scarce, and most studies used their private dataset, leading to significant variability in data sources and formats. Annotation methodologies differed across studies, with minimal shared guidelines or standards, making direct comparisons challenging. Performance metrics varied across syndromes, with F1-score, precision, and recall as the most commonly reported. Key challenges included the lack of dataset uniformity, differences in annotation practices, and the absence of external validation.

**Conclusion:**

NLP has shown potential in GS analysis, particularly for the detection of syndromes and epidemiological research. However, the majority of studies only focused on one syndrome, and variability in dataset availability, annotation processes, and model performance present challenges to broader implementation. Future research should focus on improving the comprehensiveness of GS identification, dataset standardisation, enhancing model generalisability, and integrating NLP approaches into clinical workflows.

**Supplementary information:**

The online version contains supplementary material available at 10.1186/s12911-026-03417-0.

## Background

Geriatric syndromes (GS) refer to a group of multiple health conditions that predominantly affect older adults, often arising from the complex interaction of age-related physiological changes, chronic diseases, and functional impairments. Unlike traditional disease classifications, GS do not fit within specific organ-based categories but are instead characterised by clinical syndromes such as frailty, delirium, falls, incontinence, and malnutrition  [1]. These syndromes are associated with increased morbidity, functional decline, institutionalisation, and mortality, making them critical targets for early detection, monitoring, and management  [2].

The significance of studying GS lies in their impact on the healthcare system. Older adults who experience these syndromes often require multidisciplinary interventions and long-term care strategies, putting significant demands on healthcare resources  [3]. Early identification and intervention are essential to mitigate adverse outcomes, reduce hospital admissions, and improve quality of life. However, GS are variably represented in disease ontologies, and are poorly coded in electronic healthcare records. The variability makes structured data difficult to use as a reliable ground truth for NLP tasks, because the link between clinicians’ narrative descriptions and the codes assigned to these syndromes is often poorly captured. Many GS are expressed implicitly in clinical notes rather than explicitly coded, and some codes aggregate multiple concepts or overlap with other diagnoses. These issues affect both supervised learning and evaluation, further motivating methods that automatically analyse unstructured text directly to capture information missing from structured data. This lack of representation in structured data poses challenges for researchers.

Electronic health records (EHRs) contain extensive clinical information that offers valuable insights into GS. However, although GS are commonly written about in unstructured free text data (such as discharge summaries, progress notes, and nursing notes), these are difficult to process using conventional data extraction techniques. Natural Language Processing (NLP) offers a promising solution by enabling the automated extraction and structuring of textual data for syndrome and risk identification as well as epidemiological research. NLP methods have been shown to significantly improve phenotype detection where structured codes are incomplete. For example, in a lupus nephritis study, the F1-measure rose from 0.52 (codes only) to 0.93 (with NLP) validated on an external cohort  [4]. Similarly, a COVID-19 hospitalisation study found that using clinical notes to define a patient phenotype was more accurate (AUROC 0.894) than using only structured data (AUROC 0.841)  [5]. In addition, in the identification of cerebrovascular disease cases in inpatient EHR data, NLP was more effective than the ICD code algorithm in detecting Cerebral Venous Disease (CeVD), suggesting its use for an automated EMR tool to facilitate future surveillance and longitudinal studies  [6]. These outcomes support our premise that, for GS which are variably coded and often described only in clinical narrative, NLP offers a promising solution.

Despite its potential, the application of NLP to GS presents several methodological and practical challenges. First, GS are variably documented, leading to sparsity problems  [7, 8]. Second, the ambiguity of medical terminology can introduce errors and has been investigated in several studies  [9, 10]; for example, the term “fall” may refer to a physical incident or a drop in blood pressure. Furthermore, some terms such as delirium, are used both as looser clinical descriptions of an individual’s mental state and as a more tightly defined formal diagnosis requiring the application of cognitive tests such as the Confusion Assessment Method (CAM)  [11] or the Delirium Rating Scale (DRS)-R98  [12] rather than textual descriptions, making their detection using NLP particularly challenging. Finally, existing NLP models trained on general clinical corpora may not generalise well to GS contexts due to variation and annotation inconsistencies.

Given these challenges, there is a need for a systematic synthesis of research on NLP applications for GS. This review aims to assess the current landscape of NLP methodologies for detecting and analysing GS, highlighting key applications, methodological trends, and limitations. By identifying gaps in the literature and defining future research directions, this work contributes to the development of more robust and standardised NLP approaches to improve geriatric care and decision making.

### Related work

Several systematic reviews have examined different aspects of GS, highlighting their complexity, risk factors, and clinical implications. One such review focused on defining GS and identifying four common risk factors, namely old age, cognitive impairment, functional impairment, and impaired mobility, across five syndromes: pressure ulcers, incontinence, falls, functional decline, and delirium  [1]. It focused on the need for complex models to capture interactions between these factors and called for strategic initiatives to integrate GS research into clinical practice and healthcare policy.

Another systematic review explored the impact of eight GS on survival among community-dwelling older adults, in contrast to hospitalised individuals, residents of nursing home, and disease-specific populations  [13]. The findings revealed that conditions such as multiple comorbidities, cognitive impairment, frailty, disability, malnutrition, impaired homeostasis, and chronic inflammation were significantly associated with poorer survival, particularly in younger old adults (62–74 years). However, the predictive value of these syndromes diminished in individuals aged 90 and older, suggesting that while GS play a role in survival modelling, their prognostic significance declines in extreme old age.

Systematic reviews on the application of NLP to clinical text have been conducted across various domains, showing its potential for extraction and analysing medical information from unstructured text. These reviews cover symptom extraction  [14], chronic diseases  [15], NLP applications in clinical text more broadly  [16, 17], radiology reports  [18, 19], cancer concept extraction  [20], cardiology  [21], and dentistry  [22]. Despite this extensive research, the intersection of NLP and GS remains largely unexplored.

To date, only one systematic review has specifically examined the application of NLP to GS. Osman et al. sought to bridge this gap by evaluating computational techniques for extracting and classifying GS from EHRs  [23]. The review identified 22 relevant studies covering syndromes such as sarcopenia, frailty, falls, delirium, dementia and incontinence, assessing the NLP methodologies, data sources, testing strategies, and performance metrics. While this review provided an exploration of NLP in the context of GS, it had a limited scope in terms of the breadth of syndromes considered.

In contrast, our review aims to provide a more comprehensive synthesis of NLP applications for GS by investigating the annotation processes, broadening the scope to cover twelve GS, and analysing the impact of the studies in terms of clinical applications. By addressing these gaps, our review seeks to establish a clearer understanding of the challenges and opportunities in leveraging NLP for GS in clinical practice. Furthermore, by identifying methodological trends and limitations, we aim to inform future research directions, promoting the development of standardised and generalisable NLP approaches for geriatric care.

Given the lack of a universal clinical consensus regarding the full list of GS, the selection of the twelve GS for this review followed a systematic, clinical-expertise, and data-driven approach. We first conducted a preliminary literature review to identify a comprehensive initial set of clinically relevant syndromes, which initially numbered more than twelve. We then applied a pragmatic filter using clinical judgement to select the final set of twelve on the basis of them being common and commonly impactful.

## Methods

This systematic review is conducted following the protocol registered on PROSPERO  [24]. The quality of the collected studies is evaluated in accordance with the Prediction Model Risk of Bias Assessment Tool (PROBAST)  [25], and the review is reported according to PRISMA guidance.

### Eligibility for literature inclusion and search strategy

We conducted a systematic literature search across seven databases that primarily focus on medical and healthcare-related research (CINAHL Plus, Embase, MEDLINE, PsycINFO, PubMed, Scopus, and Web of Science) and three specialising in technical and engineering research (Compendex, Inspec and the ACL Anthology). The inclusion of both medical and technical databases was intended to minimise the risk of omitting relevant studies and to ensure a balanced perspective on both the clinical and computational aspects of NLP applications in geriatric syndrome detection.

The search was conducted from the inception of each database until the end of November 2024. Although each database may have specific query syntax and indexing structures, all searches followed the same construct. Specifically, search queries were designed to include terminology related to geriatric syndromes as follows: (“fall*” OR “frail*” OR “weight loss” OR “unspecified cognitive impairment” OR “pressure injury” OR “decubitus ulcer” OR “pressure sore” OR “delirium” OR “dementia” OR “incontinence” OR “malnutrition” OR “visual impairment” OR “hearing impairment” OR “sensory impairment” OR “geriatric syndrome” OR “geriatric patient” OR “geriatric disease” OR “geriatric condition”) *AND* (“Natural Language” OR “NLP” OR “document classif*” OR “Named entit*” OR “Entit* link*” OR “Word* embedd*” OR “Doc* embedd*” OR “Text min*”). A detailed breakdown of the final search strategies for each database is provided in the Supplementary Material document Table 1.

To be eligible for inclusion, studies had to address at least one of the twelve GS of interest (falls, frailty, malnutrition, weight loss, delirium, dementia, unspecified cognitive impairment, urinary incontinence, faecal incontinence, pressure injury, visual impairment, and hearing impairment). Additionally, the study population was required to consist of older adults, as the review specifically focuses on geriatric issues. For this purpose, studies were included if the reported mean or median age of participants was 50 years or older. Studies were eligible if they were peer-reviewed studies using EHRs as their primary data source which explicitly employed NLP techniques for the extraction, classification, or analysis of information related to geriatric syndromes from unstructured clinical text.

Studies not written in English were excluded. However, studies based on corpora in other languages were included, provided that their findings were reported in English. Studies focusing exclusively on structured data, such as coded diagnoses or structured EHR fields, were excluded, unless they also reporting processing of unstructured text, as were studies which did not use EHRs (such as those using social media text or speech and voice data) as these fall outside the scope of clinical text analysis in the context of GS detection.

While no minimum document or training set size was imposed as part of the inclusion or exclusion criteria, dataset size is an important consideration when interpreting model performance. Studies were included regardless of dataset size, and this variation is taken into account in the interpretation of findings.

We registered the protocol to conduct this systematic review on PROSPERO (CRD42024592024)  [26].

### Manual review of literature

Covidence systematic reviewing software^1^ was used to deduplicate search findings from multiple databases, and for managing study selection which was in two-stages: (1) Title and abstract screening; and (2) Full-text review. In both stages, four researchers (IG, HZ, AH, FR) independently assessed the studies based on predefined inclusion criteria, ensuring that only those focusing on the processing of GS using NLP techniques were considered. Any discrepancies or disagreements during the screening process were resolved through discussion to reach consensus.

**Fig. 1:**
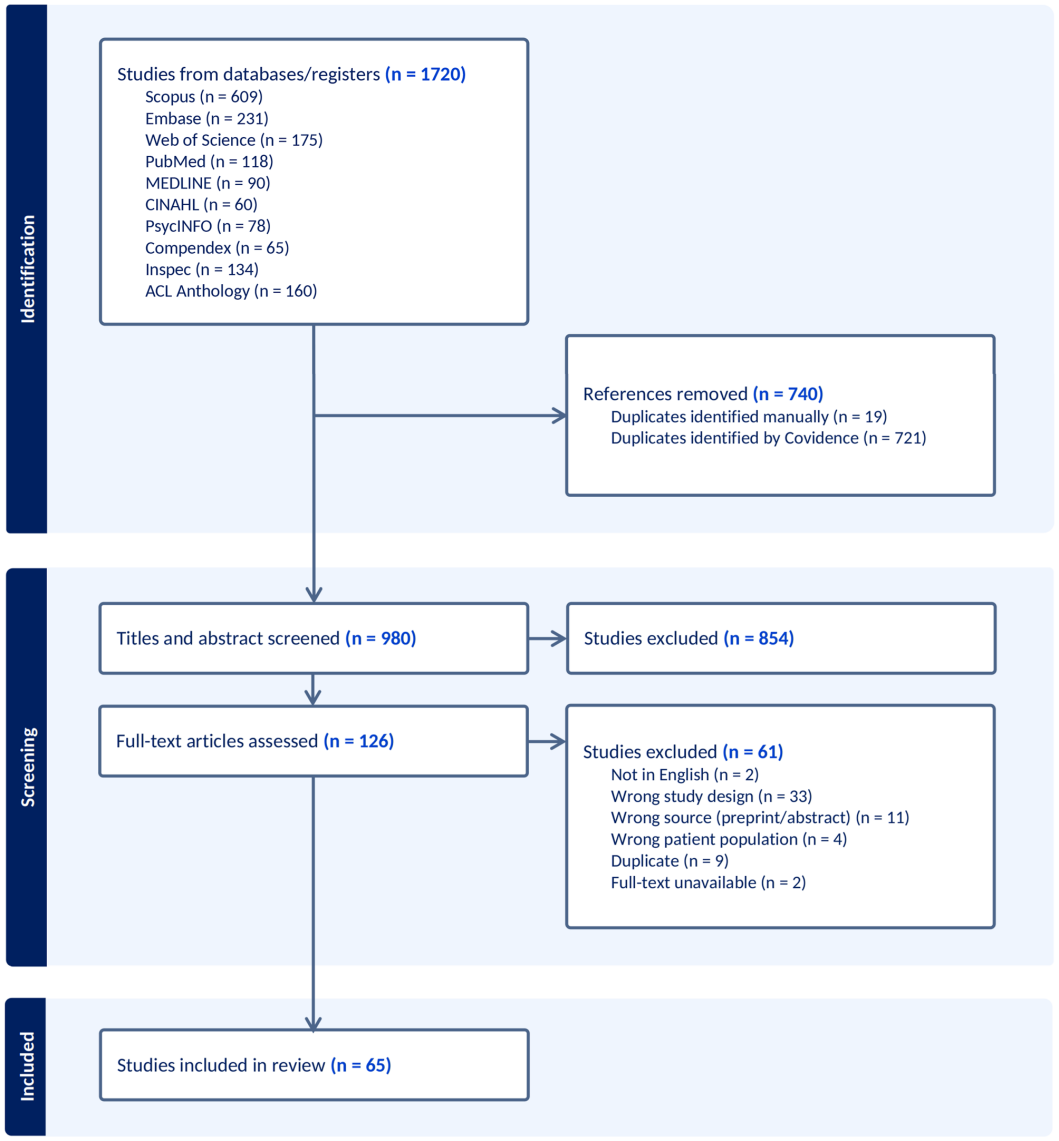
PRISMA diagram for database search

Figure 1 shows the studies selection in PRISMA chart.

### Data extraction for analysis

The data extracted for this review included the clinical application of the study, the NLP methods employed, the features or input used for NLP processing, the availability of annotation guidelines, inter-annotator agreement, data sources and their accessibility, as well as the reported NLP performance and evaluation measures. We also extracted general characteristics of the studies on the year, country, syndrome type, document type, the language of the corpus, and numerical categories such as mean age, gender ratio, and the population size. For studies reporting performance metrics, we used PROBAST to evaluate their methodological quality. PROBAST assesses the risk of bias by considering various indicators, including corpus size, study population characteristics, the generalisability of the findings, and the replicability of the proposed NLP methods.

### Clinical applications categories

We categorised the applications of the studies in this review by adapting the categories of NLP applications in radiology originally introduced by Pons et al.  [18] and subsequently refined by Casey et al.  [19]. Pons et al. initially created five categories of NLP applications: (1) diagnostic surveillance, (2) cohort building for epidemiological studies, (3) query-based case retrieval, (4) quality assessment of radiological practice, and (5) clinical support services. Casey et al. later modified them by introducing two additional categories while removing one, resulting in six categories: (1) diagnostic surveillance, (2) disease information and classification, (3) quality compliance, (4) cohort/epidemiology, (5) language discovery and knowledge structure, and (6) technical NLP.

In line with this approach, we adapted Casey et al.’s classification to the domain of geriatric syndromes, refining the categories to better reflect the nature of the included studies. Specifically, we use four application categories: (1) diagnostic surveillance, (2) syndrome information and classification, (3) cohort and epidemiology, and (4) language discovery and knowledge structure.

## Results

### General characteristics

More than half of the included studies originated from the United States of America (*n* = 36), followed by Australia (*n* = 7), Japan (*n* = 4), and Canada, China, the Netherlands, and the United Kingdom with three studies each. Brazil contributed two studies, while Italy, Spain, and Taiwan each contributed one.

There is a clear upward trend in the number of studies focusing on GS in recent years, with 12 studies in 2022, 14 in 2023, and 18 in 2024.

Table  1 shows the number of studies examining the twelve GS, with dementia, fall, and delirium as the three most frequently studied syndromes. While “lack of social support” and “walking difficulty” were not originally included in our predefined list of twelve GS, their presence among the selected studies suggests growing recognition within the field. In contrast, no studies addressed hearing impairment, highlighting a potential gap in the literature that warrants further exploration. The number of syndromes examined per study are shown in Table  2, with the majority only has one syndrome. The predominance of single-syndrome studies corresponded with the way most included papers defined and scoped their research questions. Only a small number of studies examined multiple syndromes, and those that did often required additional steps for defining and labelling multiple clinical concepts.

**Table 1:** Study counts by syndrome

Syndrome	No. studies (%)
Dementia	28 (43.08)
Fall	26 (40)
Delirium	10 (15.38)
Malnutrition	6 (9.23)
Urinary incontinence	5 (7.69)
Visual impairment	5 (7.69)
Weight loss	5 (7.69)
Faecal incontinence	4 (6.15)
Pressure injury	4 (6.15)
Unspecified cognitive impairment	4 (6.15)
Frailty	3 (4.62)
Lack of social support	3 (4.62)
Walking difficulty	3 (4.62)

**Table 2:** Number of syndromes examined per study

No. syndromes	No. studies
1	56
2	4
3	1
4+	4

The distribution of languages in the analysed corpora reveals English as the most frequent (*n* = 50), followed by Chinese (*n* = 4) and Japanese (*n* = 4), with a smaller number of other languages also represented (i.e., Dutch, Brazilian Portuguese, Italian, and Spanish).

The reported population sizes across studies vary considerably, ranging from a minimum of 23 patients to a maximum of 680,945, with a mean and median of 28,434 and 4093, respectively. Eleven studies did not report the population size. The proportion of female participants within study cohorts ranges from 10.4% to 74%, with a mean of 53.32% and a median of 55.8%. Twenty studies did not specify the proportion of female participants. The reported mean age of participants ranged from 55.4 to 85.2 years, with an overall mean of 72.8 and a median of 73.9 years. However, only 43 studies reported the mean age.

### Data origins and availability

The reviewed studies drew upon a variety of datasets from diverse origins. A significant portion of datasets originated from academic institutions (*n* = 28), highlighting the role of universities and research centres in generating and curating clinical text corpora for NLP research. Healthcare organisation contributed data (*n* = 23) demonstrating the real-world clinical practice is driving NLP research. Governmental sources (*n* = 7), including the Veteran Health Administration, and hospitals (*n* = 6) also provided data, and one study further utilised public digital libraries to develop a knowledge base for fall risk assessment tools

Several lexicons, ontologies, and external knowledge bases employed to enhance vocabularies are also reported. Among these, the Unified Medical Language System (UMLS) metathesaurus for multiple biomedical terminologies, was the most frequently utilised (*n* = 7). This was followed by the Systematized Nomenclature of Medicine - Clinical Terms (SNOMED CT), a standardised clinical terminology for medical concept mapping (*n* = 4), the International Classification of Diseases (ICD), used for the coding and categorisation of diagnoses and procedures (*n* = 3), and the International Classification for Nursing Practice (ICNP) (*n* = 3).

Despite the widespread use of clinical datasets, data accessibility remains a significant challenge. Out of the 65 studies, only one study in Brazilian Portuguese has publicly released its dataset  [27], while two others provide access upon request  [28, 29].

### Annotation and inter-annotator agreement

Few studies reported their annotation tools used in their workflows. The tools mentioned include REDCap^2^, BRAT^3^, Multi-document Annotation Environment (MAE)^4^, Visual Tagging Tool^5^, Text Mining Studio^6^, and WebAnno^7^. These varied platforms reflect the range of needs from structured data collection (e.g., REDCap) to specialised text annotation interfaces (e.g., brat and WebAnno). We attributed the absence of reported annotation tools in many studies to the lack of best practice or using simpler methods, such as tabular data in spreadsheets, which may not offer the same level of support of consistency checks and inter-annotator agreement analysis.

Table  3 shows common characteristics of the annotation reported in the studies. 17 studies reported the inter-annotator agreement (IAA) metrics with Cohen’s Kappa  [30] as the most common one to measure agreement between two annotators on categorical labels. This is followed by Fleiss’ Kappa  [31], which generalises Cohen’s Kappa for multiple annotators, and F1-score  [32], which is useful when evaluating annotation consistency in information retrieval and classification tasks. Additionally, pairwise agreement, a less common but straightforward metric, is reported by one study.

**Table 3:** Characteristics of annotation process

	No. of studies	Min	Max	Median	Note
IAA metric	17	-	-	-	Cohen’s Kappa (10), Fleiss’ Kappa (3), F1-score (3), pairwise (1)
IAA value	18	0.49	0.971	0.854	
No. annotators	33	1	7	3	
Annotation provided	7	-	-	-	

IAA values are reported in 18 studies, with scores ranging from a minimum of 0.49 to a maximum of 0.971, and a median value of 0.854. Higher IAA values indicate strong consensus among annotators, while lower ones suggest moderate or poor agreement. However, IAA may also vary depending on the annotation task complexity, dataset characteristics, or annotator expertise.

Thirty-three studies reported the number of annotators involved, ranging from a single annotator to seven, with median three. The use of a single annotator could raise concerns regarding subjectivity and potential bias. In contrast, larger annotation teams may improve reliability but require well-defined annotation guidelines to ensure consistency. However, only seven studies provided their annotation guidelines.

The resolution of annotation disagreements across the reviewed studies primarily relies on expert consultation, discussion among annotators, and adjudication by a third party. A common approach involves consulting subject matter experts, such as clinicians, domain specialists, or experienced annotators  [33, 34]. Others report resolving disagreements through discussion within annotation teams, consensus discussions, or expert panel reviews  [27, 35–39]. In some cases, adjudication is performed by a designated reviewer (or a pair of reviewers) to ensure consistency and reduce subjectivity  [40, 41]. Some studies mentioned mediation by a third-party judge or involvement of interdisciplinary experts, such as geriatricians and palliative care physicians to enhance the reliability of annotations  [42, 43].

### NLP preprocessing and input features

The most common preprocessing steps taken are shown in Table  4, with tokenisation as the most frequently reported technique to break text into meaningful units for further analysis. Text normalisation through case conversion and stop word removal is also widely adopted as a common strategy to standardise text. Further techniques such as N-gram generation, number normalisation and stemming show the effort to capture contextual relationships and standardising numerical and morphological variations. Additionally, techniques such as punctuation handling and synonym augmentation are also used to refine text quality. The ‘Other’ category consists of negation handling (*n* = 3), lemmatisation (*n* = 3), part-of-speech tagging (*n* = 3), and dependency parsing (*n* = 2). Finally, in languages that use Sino-Japanese characters, morphological analysis (*n* = 1) and characters unification (*n* = 1) are reported.

**Table 4:** Most common preprocessing steps and the number of studies

Preprocessing	No. of studies	Preprocessing	No. of studies
Tokenisation	22	Number normalisation	8
Text lowering/uppercasing	15	Stemming	8
Stop words removal	14	Punctuation handling	5
Other	13	Synonym handling	4
N-grams	8		

For preprocessing steps, the eight most commonly used tools or libraries reported in the studies are shown in Table  5. NLTK is reported as the most frequently used library, showing its longstanding role as a foundational toolkit for NLP tasks such as tokenisation, stemming and stop-word removal. MedTagger, NimbleMiner, GATE and cTAKES show the trend of using specialised frameworks tailored to extracting medical concepts from unstructured data. The use of spaCy and scispaCy shows the growing adoption of modern NLP libraries that offer efficient tokenisation, named entity recognition and syntactic parsing, particularly in clinical domain. Two studies reported the use of proprietary data mining tools such as SAS Enterprise Miner. Finally, the ‘Other’ category consists of Medical Text Extraction, Reasoning, and Mapping System (MTERM), Concept Encoder, Text Mining Studio, Moonstone, and MeCab.

**Table 5:** Most common preprocessing tools/libraries and the number of studies

Tool/library	No. of studies	Tool/library	No. of studies
NLTK	6	scispaCy	3
Other	5	General Architecture for Text Engineering (GATE)	2
MedTagger	4	clinical Text Analysis and Knowledge Extraction System (cTAKES)	2
NimbleMiner	4	SAS Enterprise Miner	2
spaCy	3		

Regarding the input vectors reported across the reviewed studies, BERT based embeddings (MIMIC based, DistilBERT, ClinicalBERT and BioClinicalBERT) are the most frequently adopted embeddings (*n* = 11), followed by Word2Vec (*n* = 7). RoBERTa and its variants (MIMIC based and MedRoBERTa.nl) are explored in several studies (*n* = 4), while fastText (*n* = 2) and GloVe (*n* = 2) demonstrate alternative word representation strategies. TF-IDF, a more traditional feature extraction method, continues to be used (*n* = 3), often in conjunction with other embeddings. Additionally, some specialised biomedical embeddings, such as BioWordVec, GatorTron, and scispaCy, are reported in a limited number of studies.

### NLP approaches in use

Table  6 summarises the frequency of NLP methods used across the reviewed studies. Rule-based approaches, including regular expressions, were the most frequently used (21 studies). The next most commonly adopted methods were transformer-based models from the BERT family (such as RoBERTa, BioClinicalBERT, GatorTron, Longformer, and BERTopic) as well as Support Vector Machine, each reported in 12 studies.

**Table 6:** Frequency of NLP methods used in the studies

Method	Count	Method	Count
Rule-based	21	Decision Tree	6
BERT	12	Tools	6
Support Vector Machine	12	Feed Forward Network	4
Logistic Regression	11	Clustering	3
Recurrent Neural Network	11	K-Nearest Neighbours	2
Boosting	10	LLMs	2
Random Forest	9	Naive Bayes	2
Other	7		

Logistic Regression and Recurrent Neural Network (including Long Short-Term Memory, Gated Recurrent Unit, and hybrid models using Convolutional Neural Network) were the next most frequently used method, each reported in 11 studies. Ensemble-based Boosting techniques (including AdaBoost, Gradient Boosting, and LightGBM) reported in 10 studies. Random Forest was used in 9 studies, while Decision Tree was reported in 6 studies. Similarly, bespoke tools such as MetaMap, NimbleMiner, NAT and GATE were used in 6 studies.

Less frequently used methods included Feed Forward Neural Network (4 studies), clustering techniques (3 studies), and K-Nearest Neighbours, large language models (LLMs), and Naive Bayes, each reported in 2 studies. The ‘Other’ category (7 studies) includes methods such as ElasticNet, Markov Chain Monte Carlo, Conditional Random Fields, hybrid and ensemble techniques as well as statistical analyses. These diverse methodologies demonstrate the heterogeneity of clinical NLP tasks, where rule-based and traditional machine learning techniques remain relevant next to modern deep learning approaches.

**Fig. 2:**
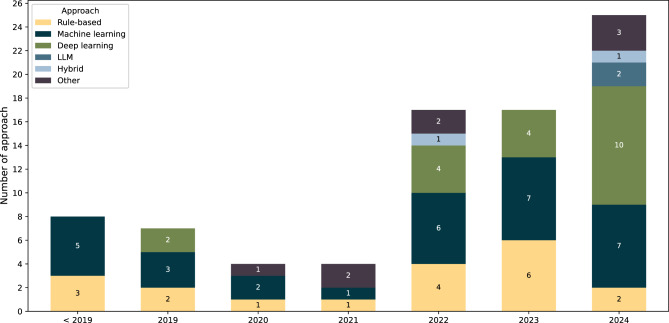
The annual trend of NLP approaches by year shows a resurgence of deep learning in recent three years, while machine learning and rule-based methods remain widely utilised. Although still in its early stages, the adoption of LLMs has shown a gradual increase over the past year

Figure 2 shows the annual trends in the adoption of NLP approaches across the studies reviewed. The numbers presented correspond to the number of times each approach is used within a study, therefore a single study may contribute to several approaches. We separated machine learning from deep learning, with the latter is for models that use neural network architectures. Additionally, pretrained language models, such as BERT, are categorised under deep learning. Studies that integrate multiple approaches within a single framework are classified as hybrid.

Over the past three years, there has been a resurgence in the use of deep learning, while machine learning methods have remained consistently utilised. In contrast, the use of rule-based approaches has shown a minor decline. Although still relatively limited, studies using LLMs appear in 2024. The ‘Other’ category includes the use of bespoke tools such as NimbleMiner, KH Coder, NLP-powered annotation tool (NAT), and statistical analysis.

The distribution of document sizes used in training for different NLP methods across the reviewed studies is summarised in Table  7. The deep learning approaches show the widest range of training size, with a minimum of 381 and a maximum of 149,655 documents. This reflects the data-hungry nature of deep learning models, where larger datasets are typically required to achieve good performance. With some studies still using a relatively small dataset, it may pose risks of overfitting. Traditional machine learning methods show a range of dataset sizes, spanning from 304 to 93,157 documents. While these methods generally require fewer labelled samples than deep learning models, they still benefit from larger training set to improve feature extraction and performance. In contrast, rule-based methods, which rely on handcrafted rules, tend to operate on smaller datasets, with a median of 779 and a maximum of 19,534. For LLMs, the document size ranges from 399 to 4749, with a median of 2574. The moderate size for LLMs is likely because the models are often pre-trained on huge corpora before being fine-tuned on smaller domain specific datasets. The hybrid methods which integrate rule-based and machine learning or deep learning as well as ensemble methods reported medium-size datasets ranging from 4357 to 4749.

**Table 7:** The document sizes for training reported in the studies

Method	Min	Max	Median
Deep learning	381	149,655	3,090.5
Hybrid	4,357	4,749	4,553
LLM	399	4,749	2,574
Machine learning	304	93,157	3,901
Rule-based	200	19,534	779

In terms of the size of training, validation, and testing datasets, a total of 31 studies reported the size of their training dataset, indicating that most studies provide details on the amount of data used to develop their models. However, only 14 studies reported the size of their validation datasets, while 24 studies reported the test datasets. Furthermore, 13 studies reported that they use cross validation technique to evaluate their approach, with the number of folds ranging from three to ten. Additionally, many studies reported the size of their cohorts but did not explicitly mention the dataset size, making it challenging to assess the exact amount of data available for model training and evaluation.

### Clinical application

**Fig. 3:**
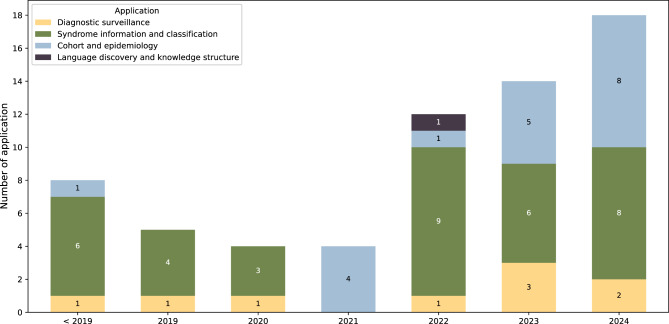
The annual trend of NLP applications by year showing “syndrome information and classification” is still prevalent every year, whilst “cohort and epidemiology” is gaining track over the past three years

Following the application categories described in Sect. 3.4, Figure 3 shows NLP applications over time. The application of syndrome information and classification is prominent throughout the period, with the exception of 2021. Meanwhile, the use of NLP for cohort and epidemiology studies has been steadily increasing, showing a growing interest in examining the characteristics of GS within populations. The exclusion of the quality compliance and technical NLP categories from Casey et al. was due to the absence of studies in our review explicitly addressing these aspects.

#### Diagnostic surveillance

Nine studies in this category focused on the automated identification of GS for surveillance purposes [44–52]. These applications aim to detect and flag the presence of specific syndromes or related clinical findings within EHRs, thereby facilitating early identification and monitoring.

Li et al. explored content selection strategies for medical notes to develop a dementia risk prediction model with a one-year prediction horizon, relying solely on textual content without using other EHR modalities  [44]. Tsai et al. adapted the click-through rate approach from commerce systems to predict dementia risk over a five-year horizon using a multimodal input of ICD-10 code embeddings and patient profiles  [45]. Topic modelling has also been leveraged, with Dormosh et al. integrating topic-based features with other EHR variables to enhance fall prediction within a one-year window  [46]. Similarly, Zolnoori et al. combined topics extracted from home healthcare data with risk factors derived from word embeddings to predict Alzheimer’s disease across a one- to four-year horizon  [47].

Embedding-based approaches have been widely applied, as demonstrated by Mishra et al., who developed a fall prediction model using word embeddings from EHR content with a two-month horizon  [48]. Kawazoe et al. utilised BERT embeddings from clinical notes to examine the impact of fall-related factors on prediction performance but found no significant improvement within a three- to thirty-day window. Their model was further extended to estimate hospital stay duration, offering insights into both patient care and financial considerations  [49]. Expanding on predefined risk factors, Hane et al. applied term similarity using fastText^8^ to predict dementia onset within a three- to eight-year horizon  [50]. In the context of hospitalisation prediction, Knapp et al. employed the Generalised Architecture for Text Engineering (GATE)^9^ to extract relevant diagnoses, enabling predictions for patients with Alzheimer’s disease within a six-month timeframe  [51]. Finally, Topaz et al. evaluated NimbleMiner^10^ for detecting and predicting fall within two-day and two-week prediction horizons, comparing its performance with a rule-based algorithm  [52].

Across these studies, minimal token or entity annotation was employed, with document embeddings or risk-related terms serving as primary features. A key methodological distinction lies in whether models relied solely on clinical text or incorporated structured patient profile data. While Knapp et al.  [51] did not explicitly develop a surveillance model for a geriatric syndrome, their approach was syndrome-driven in predicting hospitalisation risk, justifying its inclusion in this category.

#### Syndrome information and classification

Unlike diagnostic surveillance, which focuses on early detection and alerting mechanisms, studies in this category primarily address syndrome detection and characterisation without direct consideration of clinical interventions or predictive modelling. This category represents the largest proportion of studies in our review, with 36 publications dedicated to the identification and classification of GS from EHRs [27, 33–35, 39–43, 53–79]. The extracted information may contribute to standardised coding, facilitate retrospective analyses, and support the development of structured datasets for future research.

Du et al. applied prompting and k-shot learning to LLMs, with GPT-4 demonstrating better performance over Llama 2 in detecting mild cognitive impairment (MCI) from EHR data. However, neither LLM outperformed classical models such as XGBoost and attention-based deep neural networks. The most effective approach combined LLM outputs with classical models in an ensemble framework using majority voting  [33]. Ge et al. developed a model to classify fall-related injuries at the paragraph level using SVM, leveraging embeddings from BERT, RoBERTa, ClinicalBERT, and DistilBERT  [53]. Vithanage et al. applied named-entity recognition (NER) to extract symptoms of agitation in dementia patients, utilising BioClinicalBERT embeddings  [54]. A rule-based approach by Prakash et al. classified the severity of Alzheimer’s disease and related dementias (ADRD) by identifying key trigger words (e.g., “mild dementia”) in clinical text  [55]. Amjad et al. evaluated the effectiveness of different word-based features for delirium classification, finding that models trained exclusively on positive words outperformed those incorporating both positive and negative words, as well as those using TF-IDF  [56].

Considering patients with dementia, Laurentiev et al. built models to detect functional impairment, i.e., activities of daily living (ADLs) and instrumental ADLs (iADLs)  [35]. Guo et al. focused on classifying nine diseases related to visual impairment by selecting the best-performing Chinese-language NER models trained with various word embeddings  [57]. Cheligeer et al. demonstrated that a fine-tuned BERT model for Alberta hospital EHRs outperformed standard BERT, BioClinicalBERT, classical machine learning models, and ICD-10 rule-based methods in detecting inpatient falls  [34]. St. Sauver et al. used NLP to identify delirium-related concepts in EHR text, facilitating the calculation of delirium rates per 100 hospitalisations based on the number of delirium episodes per patient  [58]. Millet et al. built a fall detection model using TF-IDF features extracted from clinical text  [59]. Powell et al. classified binary falls type (falls due to perturbations to the individuals’ centre of mass (CoM) or base of support (BoS)) from self-reporting data for people in Parkinson’s Disease using features extracted from word clusters and embeddings from RoBERTa  [60].

Maclagan et al. built dementia detection using TF-IDF as the features from the consult notes, progress notes, and the combination of both  [61]. A focus on cohort-specific modelling was explored by Shao et al., who applied topic modelling to dementia detection by developing separate models for Black American and White American cohorts. Their findings indicated that using distinct models improved detection performance in the Black American cohort, highlighting the significance of cohort selection in model development  [41]. Liu et al. used BioClinicalBERT for dementia classification, experimenting with both binary (yes/no) and ternary (yes/no/uncertain) labels. Their results revealed substantially better performance in the binary classification task  [62]. Penfold et al. detected MCI using 42 concepts detected using NLP. Although their main aim is to detect MCI, the 42 concepts can be used as epidemiological study for MCI.  [63]. Using ambulance EHRs, Tohira et al. built fall detection models using top-n TF-IDF as the inputs  [64].

In the absence of routine delirium screening, Chen et al. proposed a rule-based model based on a Chinese chart-based keyword scale  [39]. Using rule-based, classical and context-aware word embeddings, Fu et al. found that a hybrid post-hoc rule for context-aware word embeddings output gives a better performance to detect falls. This approach further adds interpretability to the model output  [65]. Wang et al. used machine learning models with TF-IDF, word count, and n-grams as inputs to detect delirium in hospital inpatients  [66]. Additionally, Fu et al. developed two rule-based delirium detection algorithms, one using a binary classification (yes/no) and another using a third category (yes/no/possible), based on NLP-detected Confusion Assessment Method (CAM) terms  [43]. Beyond detection models, Noori et al. evaluated the NLP-powered annotation tool (NAT) for cognitive status phenotyping, reporting high interrater agreement and demonstrating its ability to expedite annotation processes  [42].

Ge et al. assessed sentence-level delirium detection models against ICD coding, demonstrating the better performance of BERT-based embeddings  [67]. In a study using Japanese EHRs, Nakatani et al. applied Markov Chain Monte Carlo (MCMC) methods to predict inpatient falls, varying factors such as data duration before a fall (imminent/not imminent) and patient stay duration  [68]. For detecting ten GS in clinical text, Kharrazi et al. developed a rule-based model  [69], which was later expanded by Chen et al. using a conditional random field (CRF) approach, incorporating varied linguistic features  [70]. Further improvements were made by considering contextual information from surrounding sentences, entire documents, and diagnosis codes using neural networks  [71].

Moreira et al. integrated structured EHR data with term clusters derived from text mining to enhance dementia detection models  [72]. Recognising that falls are often documented using a limited set of standard phrases, Patterson et al. designed a rule-based system for detecting falls in emergency department notes  [73]. An ontology-based approach was adopted by Zeng-Treitler et al., who developed a frailty extraction model to calculate frailty scores, which were then used with other features to predict post-surgical mortality  [74]. In another study on Japanese clinical text, Toyabe applied syntactic rules to progress notes, discharge summaries, image order entries, and incident reports, identifying incident reports and image order entries as the most informative sources for fall detection  [75]. Two earlier studies by Tremblay et al. explored fall detection by incorporating term clusters from latent semantic indexing  [76] and terms selected using entropy weighting and information gain  [77].

In home healthcare settings, Topaz et al. developed a bespoke tool to identify six neuropsychiatric symptoms of ADRD and their related terms in clinical notes  [40]. Using twelve different word embeddings, dos Santos et al. compared and evaluated their efficacy against TF-IDF for fall detection  [27]. For delirium detection in COVID-19 patients, Pagali et al. benchmarked a previously developed rule-based algorithm  [43] against ICD coding and nursing assessments, finding it achieved higher diagnostic sensitivity  [78]. Within frailty research, Martin et al. trained models to identify four aspects of frailty at the sentence level, leveraging Word2Vec, BioClinicalBERT, and RoBERTa embeddings  [79].

#### Cohort and epidemiology

This category includes studies that utilise NLP to extract and analyse information on the prevalence, distribution, and associations of GS within clinical populations. In this review, 19 studies focused on the identification of GS and their patterns within healthcare data [28, 29, 36–38, 80–93]. These studies contribute to a broader understanding of syndrome burden, risk factors, and trends over time, thereby informing clinical guidelines and healthcare policy. Although this category was adapted from previous frameworks by Pons et al. and Casey et al., we only identified studies retrospectively addressing epidemiological analyses rather than prospective cohort construction.

Gibson et al. examined the characteristics of individuals with very late-onset psychosis that may progress to dementia with Lewy bodies, identifying schizophrenia, delusional disorder, acute psychotic disorder, nonorganic psychosis, and schizoaffective disorder as the most prevalent conditions  [80]. The evolution of topics associated with falls over a three-year period was analysed by Dormosh et al. using topic modelling, revealing 264 topics, with 25 displaying significantly different trends between case and control groups  [81]. Panahi et al. identified seven frontotemporal dementia (FTD) related symptoms and features in post-9/11 era veterans’ clinical notes  [28]. Within the context of Alzheimer’s disease, Sivarajkumar et al. explored sleep-related patterns, extracting seven distinct patterns using rule-based methods, machine learning, and LLMs. Among these approaches, rule-based techniques and a fine-tuned LLaMA 2 model demonstrated strong performance  [36]. Investigating the causes of falls in hospital inpatients, Zhang et al. utilised KH Coder^11^ to identify sixteen text clusters, covering factors related to patient conditions, excretion activities, and dynamic interactions between patients, objects, and caregivers in their environment  [37].

Chen et al. extracted eight delirium symptoms using NER with five transformer-based models  [82]. Ryvicker et al. leveraged NimbleMiner to identify signs and symptoms of ADRD in home healthcare patients  [83], while Scharp et al. also used NimbleMiner to analyse the symptoms of urinary incontinence, exploring differences in documentation by race or ethnicity  [84]. NLP applications in Sino-Japanese clinical text were highlighted in a study by Miyazawa et al., who used n-grams to classify risk factors for delirium onset in Covid-19 patients  [85]. Wu et al. applied rule-based methods to identify seven social determinants of health in ADRD patients, further demonstrating the potential of NLP in extracting relevant clinical insights  [38].

Oh et al. employed text mining to extract ADRD-related clinical phenotypes, encompassing medical comorbidities, biomarkers, neurobehavioural test scores, behavioural cognitive decline, family history, and neuroimaging findings  [86]. Rather than working directly with EHRs, Altuhaifa et al. constructed fall risk factors from fall risk assessment tools and later used these factors to identify their presence in clinical data  [87]. Chen et al. developed a rule-based NLP pipeline to extract cognitive tests and biomarkers, categorising them into severity levels  [88]. Alkhalaf et al. identified 15 causative factors for malnutrition in aged care facility patients using rule-based extraction techniques  [89]. Focusing on intensive care settings, Young et al. investigated delirium-related behavioural disturbances in critically ill patients, further assessing their impact on ICU mortality, ICU length of stay, and overall hospital stay duration  [90].

Lorenzoni et al. examined whether incorporating topic clusters derived from latent Dirichlet allocation (LDA) into coded EHR information could improve the characterisation of in-hospital falls  [29]. The relationship between geriatric syndromes and frailty was analysed by Anzaldi et al. using pattern-based matching, revealing that frailty commonly co-occurs with walking difficulty, lack of social support, falls, and weight loss  [91]. Zhou et al. identified twenty lifestyle exposures among ADRD patients using MetaMap^12^ and UMLS^13^, categorising them into dietary factors, daily activities, and substance abuse  [92]. Investigating the consequences of weight loss, Soysal et al. assessed its impact on hospitalisation and mortality in patients with Alzheimer’s disease, vascular dementia, and dementia with Lewy bodies. Their findings indicate that while increased mortality is specifically associated with Alzheimer’s disease, hospitalisation risk is linked to all three dementia types  [93].

#### Language discovery and knowledge structure

This category includes studies that examine the linguistic characteristics of clinical text for understanding how variations in clinical documentation affect the automated identification of syndromes. Only one study in this review fell into this category. A pilot study conducted by Leurs et al. to examine linguistic patterns in clinical documentation related to falls, focusing on word-level and n-gram analysis within Dutch nursing reports. Their findings indicate that certain high-frequency unigrams are uniquely prevalent in the documentation of patients who have experienced a fall  [94].

#### Quality and compliance

From Pons et al. and Casey et al., this category covers studies that utilise NLP to assess the quality, consistency, and adherence of clinical documentation to established guidelines and standards. Such applications can support internal quality assurance, enhance patient safety, and provide insights into documentation practices related to geriatric syndromes. No studies in this review addressed these aspects.

#### Technical NLP

From Casey et al., this category includes studies primarily focused on methodological advancements in NLP, rather than the direct extraction or analysis of domain specific information. This may include techniques such as negation detection, spelling correction, fact checking, or optimising annotation processes in EHRs for GS. No studies in this review addressed these aspects.

### NLP performance and evaluation measures

Assessing the overall performance of the reviewed studies through a formal meta-analysis is challenging due to the heterogeneity of tasks and the evaluation metrics employed. The studies covers a diverse range of NLP tasks, including classification that can be further specialised into binary and multiclass classification, while others did statistical analysis or clustering and language characteristic observation. Some studies reported standard classification metrics such as precision (positive predictive value, PPV), recall (sensitivity or true positive rate, TPR), and F1-score (the harmonic mean between precision and recall). Other metrics such as Area Under the Receiving Operating Characteristic Curve (ROC AUC) for binary classification, specificity (true negative rate, TNR), and negative predictive value (NPV) were also used.

**Fig. 4:**
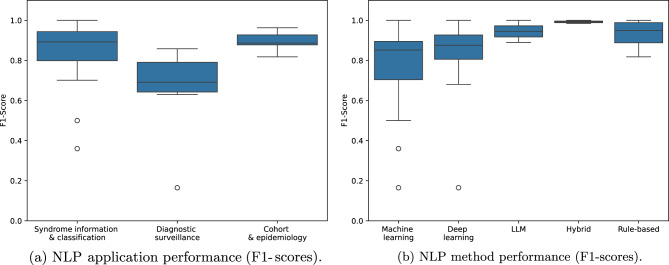
Comparison of NLP performance across applications and methods based on reported F1-scores

To address this issue, we compiled only the studies that reported the F1-score or reported data from which it could be calculated (e.g., a confusion matrix), as it is the most prevalent score used in the reviewed studies. Although this does not allow for a direct comparison of performance across studies, given the diversity of tasks addressed, it serves as an exercise to provide a general overview of NLP performance within this domain. If a study evaluated multiple methods, we selected the best performing approach as reported. Figure 4a and Figure 4b present box plots of the F1-score distribution across application categories and NLP methods, respectively. In Figure 4a, only three categories are presented, as the only study in the language discovery and knowledge structure category did not provide performance metrics, instead focusing on linguistic characteristics of clinical notes. The diagnostic surveillance category shows a lower mean and median F1-score compared to other applications, which may reflect the complexity and variability of diagnostic prediction tasks in clinical settings.

Rule-based methods obtained the highest mean and median F1-score compared to other methods, suggesting that manually crafted rules continue to perform robustly in clinical NLP tasks, particularly in tasks with consistent linguistic patterns or smaller, balanced datasets. A key factor underlying this performance is the iterative refinement of rules and lexicons, in which initial patterns are applied to annotated training data, errors are manually reviewed, and rules are systematically adjusted to handle false positives, false negatives, negations, and domain-specific variations. Unlike machine or deep learning approaches, which rely on hyperparameter tuning or larger datasets for performance improvement, rule-based systems achieve gains through careful, task-specific adjustments. While this process enhances interpretability and task-specific accuracy, it may limit scalability and generalisability. The performance of hybrid and LLM methods is uncertain due to their limited representation in the literature to date.

While the F1-score provides a useful measure of model performance, it is important to consider the underlying class distributions in the datasets. Class imbalance is a significant methodological concern as some conditions are much more prevalent in EHRs than others. Several studies explicitly reported strategies to mitigate this challenge, which predominantly fell into three main categories: data-level, algorithmic-level, and evaluation/metric-level approaches.

At the data level, which involves modifying the composition of the training data, some studies used sampling methods (oversampling or downsampling) to balance class representation  [44, 45, 68, 72, 76]. Other data-level approaches included targeted data curation to ensure balanced representation across subgroups  [53] and feature enrichment, such as lexicon creation, to increase the prevalence of relevant minority-class examples  [35].

At the algorithmic level, which alters the learning process during training, three studies used methods to assign greater importance to the minority class, such as using the inverse of class frequency  [49], assigning explicit weights to minority labels  [48], or using weighted classifiers  [60].

At the evaluation/metric level, which focuses on providing an unbiased performance assessment in the presence of imbalance, several studies specifically used the micro, macro, or weighted F1-score to account for class imbalance and provide a balanced assessment of performance across classes  [36, 60, 61, 66, 67, 71]. Others tuned classification thresholds or performed cut-off optimisation  [34, 47], allowing models to be adapted to specific clinical goals (e.g., prioritising high sensitivity versus high positive predictive value) despite data imbalance.

Despite these strategies, it is notable that many primary studies did not explicitly report how they addressed class imbalance. This represents an important methodological gap, as class imbalance can substantially affect model performance, evaluation, and generalisability.

### Quality assessment

We conducted a quality assessment of the reviewed studies using the PROBAST criteria to evaluate the robustness of the approaches taken and potential biases. Overall, most studies followed rigorous methodologies in their research, with some notable exceptions.

In terms of cohort size and participant numbers, nine studies were assessed as having a high risk of bias  [27, 52, 56, 57, 60, 75–77, 94], while the risk of bias was unclear in four studies  [49, 54, 64, 66]. Specifically, the pilot study in [94] included only 19 patients and 19 controls [60], examined 23 individuals [56], studied 54 patients, and [57] involved 60 participants. Although these studies acknowledged their limited sample sizes, small cohorts can lead to overfitting in machine learning models and reduce the generalisability of their findings. Furthermore [27, 52, 75–77], did not explicitly state the population’s age in their study demography. Additionally [54], reported only the total number of clinical notes used without it being clear if this figure also reflected the cohort size. Similarly [49, 64], reported only the number of fall cases, while [66] provided the number of admission without specifying the patient count.

In terms of predictor and outcome definition, two studies were assessed as having a high risk of bias. [33] did not split the training and testing datasets based on patients. This means a patient could have some of their clinical notes in the training set and others in the test set, introducing data leakage as the pattern of that particular patient would have been recognised by the model. This can lead to overly optimistic results that may not generalise well to unseen data. However, it is worth noting that in some trusted research environments (TREs), researchers may not have access to patient level identifiers, making patient level splitting infeasible. Finally [87], used expert generated test data, which is likely to introduce bias, as the experts may have known what is the model trying to solve.

## Discussion and future directions

### NLP methods in GS and clinical applications

NLP has been increasingly applied to the study of GS, demonstrating its potential to extract, classify, and analyse unstructured clinical text for syndrome detection and patient monitoring. Dementia and falls were the most frequently studied syndromes, with other important GS like incontinence less commonly examined, and hearing impairment never examined. The limited attention to incontinence warrants critical reflection, as it can profoundly affect an older person’s self-esteem and restrict engagement in everyday and social activities.

In terms of NLP methods, the reviewed studies used a wide range of techniques, from rule-based to machine learning and deep learning models. While rule-based methods remain widely used due to their interpretability, machine learning and deep learning methods, particularly those using BERT and other transformer-based architectures, have gained track. The adoption of LLMs remains limited, which is likely due to computational constraints and concerns regarding privacy and hallucination. These challenges are particularly common in clinical contexts, where research tends to lag behind mainstream NLP developments by one or two years. Nonetheless, with ongoing advancements in LLMs, their use in clinical NLP is expected to grow in 2025 and beyond

The reviewed studies primarily focused on four broad application categories, namely diagnostic surveillance, syndrome information and classification, cohort and epidemiology, and language discovery and knowledge structure. Among these, syndrome information and classification was the predominant focus, reflecting an aim to facilitate automatic syndrome detection. While these efforts enhance the representation of geriatric syndromes in clinical data, further work is needed to integrate such classification approaches into practical healthcare applications and decision-support systems.

Most included studies examined a single syndrome, and only a few addressed multiple syndromes simultaneously. This predominance of single-syndrome studies highlights a current limitation in coverage, as older adults frequently have multiple co-occurring conditions and syndromes. Work outside the scope of this review illustrates that broader approaches are feasible: one recent study applied NLP methods to all twelve predefined GS within a unified framework  [95]. This study demonstrates the potential for multi-syndrome detection and classification, providing a model for future research that aims to capture the complexity of geriatric patient presentations.

### Study heterogeneity and reporting standards

A key finding in this review is the heterogeneity across studies in terms of datasets, annotation process, and evaluation metrics, which limits the ability to aggregate findings. This challenge is not unique to GS and has also been observed in other domains, such as radiology  [19] or cardiology  [21]. The lack of standardised datasets, in particular, hinders the reproducibility and generalisability of research in this area. While some studies relied on institutional or proprietary data sources, only one dataset was publicly available, limiting the opportunity for benchmarking and comparative analysis. Given the sensitive nature of clinical data, building shared repositories for anonymised clinical text remains a challenge.

To address these barriers, greater investment in data sharing initiatives and the development of accessible, well-curated datasets is essential. A number of examples illustrate the potential for enabling reproducible and generalisable research through secure access resources. These include consented datasets such as Generation Scotland, a population and family based cohort linking genetic, health, and lifestyle data with the National Health Service (NHS) EHRs to support research into diverse health conditions  [96]; MIMIC-III  [97] and its successor MIMIC-IV  [98], large, deidentified clinical databases containing detailed hospital-wide and ICU specific data, such as vital signs, medications, lab results, microbiology cultures, clinical notes, and imaging reports, from tens of thousands of critical care patients; The Scottish Medical Imaging (SMI) Archive which offers population based radiology images linked to routine health records, enabling AI research and validation within the Scottish National Safe Haven  [99]; Brain Imaging, a large system national cohort linking brain imaging data with health system records in Scotland  [100]; and a whole population cohort dataset for investigating covert cerebrovascular disease and its neurological risk  [101]. These examples, particularly those based in Scotland, demonstrate the potential of well curated, large scale, linkable datasets to advance reproducible research, positioning Scotland as a leader in population based data access for clinical NLP in the UK.

Annotation inconsistencies further compound the challenge of standardisation, with substantial variation observed in annotation tools, inter-annotator agreement measures, and disagreement resolution strategies. While some studies used structured annotation guidelines and consensus adjudication, others lacked explicit documentation of their annotation protocols. Given that clear guidelines are essential for minimising ambiguity and ensuring reproducibility, transparency in reporting annotation procedures would enhance the reliability and comparability of results across studies. Developing best practices for annotating GS would be beneficial in enhancing consistency of clinical NLP research.

Evaluation techniques also varied between studies, making direct comparisons of model performance difficult. Although this phenomenon depends on the research tasks themselves, such as classification or extraction, how many labels being used, a standard between tasks will make comparison and analysis to be more feasible. The development of reporting guidelines similar to CONSORT for clinical trials  [102] or STARD for diagnostic accuracy studies  [103] could help address these issues and promote transparency in NLP research in GS.

### Progressing NLP in GS

While NLP research in GS has primarily focused on detecting, extracting, and predicting syndromes from clinical text, recent advances in LLMs, chatbots, and speech processing suggest new avenues for improving patient care. Beyond information extraction, NLP applications are now being developed to support real-time monitoring, conversational agents for older adults, and assistive technologies tailored to individuals with conditions such as dementia, e.g.,   [104–106]. Additionally, speech processing techniques have been used in detecting early cognitive decline by analysing speech patterns and linguistic markers, offering a non-invasive method for neurodegenerative conditions, e.g.,   [107–110].

These developments show a shift towards patient-centred NLP applications that go beyond structured text analysis to interactive systems. Future research should also explore the integration of multimodal data, including speech, computer vision, and textual records, to develop AI solutions for geriatric care, as shown in some studies that combine text and speech processing to detect dementia, e.g.,   [100, 111–113].

### Strengths and limitations of this review

This review has several key strengths. First, it used a systematic search strategy across multiple databases covering both clinical and technical domains to ensure broad coverage of relevant literature at the intersection of GS and NLP. The screening and full-text selection processes were independently conducted by multiple reviewers, reducing the risk of selection bias. Beyond mapping clinical applications and NLP methods, the review gives particular attention to the reproducibility and transparency of included studies, specifically through the examination of dataset availability, annotation protocols, and inter-annotator agreement. By highlighting annotation practices and reporting standards, this review aims to the advancement of more robust and reproducible methods in clinical NLP for GS.

#### Limitations of this review

Despite these strengths, a number of limitations should be acknowledged. Although the search strategy was comprehensive, studies published in languages other than English may have been excluded. However, this limitation was mitigated by the inclusion of English-language publications that analysed non-English clinical corpora.

In addition, substantial methodological heterogeneity, particularly in dataset size, annotation approaches, and evaluation metrics, limits direct comparisons of model performance. Rather than being a flaw of individual studies, this reflects broader challenges in the field and reinforces the need for standardised reporting and benchmarking practices.

#### Limitations in the existing literature

The included studies also exhibited several common limitations that affect the broader field. Reproducibility and generalisability remain significant challenges. Most models were evaluated using internal train-test splits, with limited external validation, raising uncertainty regarding their robustness in other clinical contexts or healthcare systems. This is closely tied to the restricted availability of clinical free-text data due to privacy concerns. In addition, many NLP workflows lack interoperability across computing environments, which can limit reproducibility and adoption. Approaches such as containerisation technologies (e.g., Docker) can help address this issue by enabling code to run consistently across different operating systems and platforms. Addressing this limitation will require both methodological advances (e.g., data deidentification), and broader support for initiatives enabling responsible data sharing and validation. Resources such as the MIMIC databases and large-scale, linkable cohorts in Scotland (e.g., Generation Scotland and the Scottish Medical Imaging Archive) illustrate how secure infrastructures can enable reproducible clinical NLP research while safeguarding patient confidentiality.

### Conclusions

This paper systematically reviews how NLP has been applied to GS, highlighting key application areas, methodological trends, as well as challenges in the field. The findings indicate that syndrome information and classification remain the predominant research focus, with deep learning and transformer based models increasingly being used alongside rule-based and traditional machine learning techniques. Most studies examined a single syndrome, and only a few addressed multiple syndromes, highlighting the limitation in coverage as older adults frequently have multiple co-occurring syndromes. Furthermore, there is still an issue in reproducibility and comparability across studies due to the substantial diversity in datasets, annotation methods and evaluation metrics, and the lack of shareable or publicly available data.

As researches in NLP advance, efforts should be directed toward developing standardised annotation guidelines, improving data accessibility, and integrating multimodal approaches. Future research should also consider scalable, interpretable, and clinically relevant applications to bridge technological innovations and geriatric care. Establishing collaborative frameworks for benchmarking and data sharing, while not ignoring the issue of ethical and sensitivity of clinical datasets, will be essential to ensure the progress and impact of NLP in GS research.

## Electronic supplementary material

Below is the link to the electronic supplementary material.

Supplementary Material 1

Supplementary Material 2

## Data Availability

Data is provided within the manuscript or supplementary information files.

## Footnotes

1 https://www.covidence.org/

2 https://project-redcap.org/

3 https://brat.nlplab.org/

4 http://keighrim.github.io/mae-annotation/.

5 https://lhncbc.nlm.nih.gov/LSG/Projects/Vtt/web/index.html

6 http://www.msi.co.jp/tmstudio/.

7 https://webanno.github.io/webanno/

8 https://fasttext.cc/

9 https://gate.ac.uk/

10 https://github.com/mtopaz/NimbleMiner

11 https://khcoder.net/en/

12 https://lhncbc.nlm.nih.gov/ii/tools/MetaMap.html

13 https://www.nlm.nih.gov/research/umls/index.html
