# Supplementary material for: Natural language processing for geriatric syndromes: a systematic review of methods, applications, and challenges
Source: BMC Med Inform Decis Mak. 2026 Mar 12;26:128. doi: 10.1186/s12911-026-03417-0 (PMC13097797; doi:10.1186/s12911-026-03417-0)
Supplement: Supplementary file 2 — Supplementary Material 2 [file 12911_2026_3417_MOESM2_ESM.docx]

**Table 1 Search strategy on 10 databases**

| Database | Search strategy |
| --- | --- |
| CINAHL Plus | TI (((fall* OR frail* OR "weight loss" OR "unspecified cognitive impairment" OR "pressure injury" OR "decubitus ulcer" OR "pressure sore" OR delirium OR dementia OR incontinence OR malnutrition OR "visual impairment" OR "hearing impairment" OR "sensory impairment" OR "geriatric syndrome" OR "geriatric patient" OR "geriatric disease" OR "geriatric condition") AND ("Natural Language" OR "NLP" OR "document classif*" OR "Named entit*" OR "Entit* link*" OR "Word* embedd*" OR "Doc* embedd*" OR "Text min*"))) OR AB (((fall* OR frail* OR "weight loss" OR "unspecified cognitive impairment" OR "pressure injury" OR "decubitus ulcer" OR "pressure sore" OR delirium OR dementia OR incontinence OR malnutrition OR "visual impairment" OR "hearing impairment" OR "sensory impairment" OR "geriatric syndrome" OR "geriatric patient" OR "geriatric disease" OR "geriatric condition") AND ("Natural Language" OR "NLP" OR "document classif*" OR "Named entit*" OR "Entit* link*" OR "Word* embedd*" OR "Doc* embedd*" OR "Text min*"))) AND TX (("geriatric*" or "old*" or "elder*")) |
| Embase | 1.(fall* OR frail* OR "weight loss" OR "unspecified cognitive impairment" OR "pressure injury" OR "decubitus ulcer" OR "pressure sore" OR delirium OR dementia OR incontinence OR malnutrition OR "visual impairment" OR "hearing impairment" OR "sensory impairment" OR "geriatric syndrome" OR "geriatric patient" OR "geriatric disease" OR "geriatric condition").mp. [mp=title, abstract, heading word, drug trade name, original title, device manufacturer, drug manufacturer, device trade name, keyword heading word, floating subheading word, candidate term word]  2.("Natural Language" or "NLP" or "document classif*" or "Named entit*" or "Entit* link*" or "Word* embedd*" or "Doc* embedd*" or "Text min*").mp. [mp=title, abstract, heading word, drug trade name, original title, device manufacturer,  drug manufacturer, device trade name, keyword heading word, floating subheading word, candidate term word]  3. ("geriatric*" or "old*" or "elder*").af.  4. 1 and 2 and 3 |
| MEDLINE | 1. (fall* OR frail* OR "weight loss" OR "unspecified cognitive impairment" OR "pressure injury" OR "decubitus ulcer" OR "pressure sore" OR delirium OR dementia OR incontinence OR malnutrition OR "visual impairment" OR "hearing impairment" OR "sensory impairment" OR "geriatric syndrome" OR "geriatric patient" OR "geriatric disease" OR "geriatric condition").mp. [mp=title, abstract, heading word, drug trade name, original title, device manufacturer, drug manufacturer, device trade name, keyword heading word, floating subheading word, candidate term word]  2. ("Natural Language" or "NLP" or "document classif*" or "Named entit*" or "Entit* link*" or "Word* embedd*" or "Doc* embedd*" or "Text min*").mp. [mp=title, abstract, heading word, drug trade name, original title, device manufacturer, drug manufacturer, device trade name, keyword heading word, floating subheading word, candidate term word]  3. ("geriatric*" or "old*" or "elder*").af.  4. 1 and 2 and 3 |
| PsycINFO | 1. (fall* OR frail* OR "weight loss" OR "unspecified cognitive impairment" OR "pressure injury" OR "decubitus ulcer" OR "pressure sore" OR delirium OR dementia OR incontinence OR malnutrition OR "visual impairment" OR "hearing impairment" OR "sensory impairment" OR "geriatric syndrome" OR "geriatric patient" OR "geriatric disease" OR "geriatric condition").mp. [mp=title, abstract, heading word, drug trade name, original title, device manufacturer, drug manufacturer, device trade name, keyword heading word, floating subheading word, candidate term word]  2. ("Natural Language" or "NLP" or "document classif*" or "Named entit*" or "Entit* link*" or "Word* embedd*" or "Doc* embedd*" or "Text min*").mp. [mp=title, abstract, heading word, drug trade name, original title, device manufacturer, drug manufacturer, device trade name, keyword heading word, floating subheading word, candidate term word]  3. ("geriatric*" or "old*" or "elder*").af.  4. 1 and 2 and 3 |
| PubMed | ((fall*[Title/Abstract] OR frail*[Title/Abstract] OR "weight loss"[Title/Abstract] OR "unspecified cognitive impairment"[Title/Abstract] OR "pressure injury"[Title/Abstract] OR "decubitus ulcer"[Title/Abstract] OR "pressure sore"[Title/Abstract] OR delirium [Title/Abstract] OR dementia[Title/Abstract] OR incontinence[Title/Abstract] OR malnutrition[Title/Abstract] OR "visual impairment"[Title/Abstract] OR "hearing impairment"[Title/Abstract] OR "sensory impairment"[Title/Abstract] OR "geriatric syndrome"[Title/Abstract] OR "geriatric patient"[Title/Abstract] OR "geriatric disease"[Title/Abstract] OR "geriatric condition"[Title/Abstract]) AND ("Natural Language"[Title/Abstract] OR "NLP"[Title/Abstract] OR "document classif*"[Title/Abstract] OR "Named entit*"[Title/Abstract] OR "Entity link*"[Title/Abstract] OR "Word embedd*"[Title/Abstract] OR "Document embedd*"[Title/Abstract] OR "Text mini*"[Title/Abstract]) AND ("geriatric*" or "old" OR "olde*" or "elder*") |
| Scopus | TITLE-ABS-KEY((fall* OR frail* OR "weight loss" OR "unspecified cognitive impairment" OR "pressure injury" OR "decubitus ulcer" OR "pressure sore" OR delirium OR dementia OR incontinence OR malnutrition OR "visual impairment" OR "hearing impairment" OR "sensory impairment" OR "geriatric syndrome" OR "geriatric patient" OR "geriatric disease" OR "geriatric condition") AND ("Natural Language" OR "NLP" OR "document classif*" OR "Named entit*" OR "Entit* link*" OR "Word* embedd*" OR "Doc* embedd*" OR "Text min*")) AND (ALL("geriatric*" or "old*" or "elder*")) |
| Web of Science | TI=(((fall* OR frail* OR "weight loss" OR "unspecified cognitive impairment" OR "pressure injury" OR "decubitus ulcer" OR "pressure sore" OR delirium OR dementia OR incontinence OR malnutrition OR "visual impairment" OR "hearing impairment" OR "sensory impairment" OR "geriatric syndrome" OR "geriatric patient" OR "geriatric disease" OR "geriatric condition") AND ("Natural Language" OR "NLP" OR "document classif*" OR "Named entit*" OR "Entit* link*" OR "Word* embedd*" OR "Doc* embedd*" OR "Text min*"))) OR AB=(((fall* OR frail* OR "weight loss" OR "unspecified cognitive impairment" OR "pressure injury" OR "decubitus ulcer" OR "pressure sore" OR delirium OR dementia OR incontinence OR malnutrition OR "visual impairment" OR "hearing impairment" OR "sensory impairment" OR "geriatric syndrome" OR "geriatric patient" OR "geriatric disease" OR "geriatric condition") AND ("Natural Language" OR "NLP" OR "document classif*" OR "Named entit*" OR "Entit* link*" OR "Word* embedd*" OR "Doc* embedd*" OR "Text min*"))) AND ALL=("geriatric*" or "old*" or "elder*") |
| Compendex | ((((fall* OR frail* OR "weight loss" OR "unspecified cognitive impairment" OR "pressure injury" OR "decubitus ulcer" OR "pressure sore" OR delirium OR dementia OR incontinence OR malnutrition OR "visual impairment" OR "hearing impairment" OR "sensory impairment" OR "geriatric syndrome" OR "geriatric patient" OR "geriatric disease" OR "geriatric condition") AND ("Natural Language" OR "NLP" OR "document classif*" OR "Named entit*" OR "Entit* link*" OR "Word* embedd*" OR "Doc* embedd*" OR "Text min*")) WN KY) AND (("geriatric*" or "old*" or "elder*") WN ALL)) |
| Inspec | ((((fall* OR frail* OR "weight loss" OR "unspecified cognitive impairment" OR "pressure injury" OR "decubitus ulcer" OR "pressure sore" OR delirium OR dementia OR incontinence OR malnutrition OR "visual impairment" OR "hearing impairment" OR "sensory impairment" OR "geriatric syndrome" OR "geriatric patient" OR "geriatric disease" OR "geriatric condition") AND ("Natural Language" OR "NLP" OR "document classif*" OR "Named entit*" OR "Entit* link*" OR "Word* embedd*" OR "Doc* embedd*" OR "Text min*")) WN KY) AND (("geriatric*" or "old*" or "elder*") WN ALL)) |
| ACL Anthology  (Regular expression using python API) | (?=.*((?=.*(fall\w*\|frail\w*\|weight\ loss\|unspecified\ cognitive\ impairment\|pressure\ injury\|decubitus\ ulcer\|pressure\ sore\|delirium\|dementia\|incontinence\|malnutrition\|visual\ impairment\|hearing\ impairment\|sensory\ impairment\|geriatric\ syndrome\|geriatric\ patient\|geriatric\ disease\|geriatric\ condition))(?=.*(Natural\ Language\|NLP\|document\ classif\w*\|Named\ entit\w*\|Entit\w*\ link\w*\|Word\w*\ embedd\w*\|Doc\w*\ embedd\w*\|Text\ min\w*))))(?=.*(age\w*\|geriatric\w*\|elder\w*\|old\w*)) |


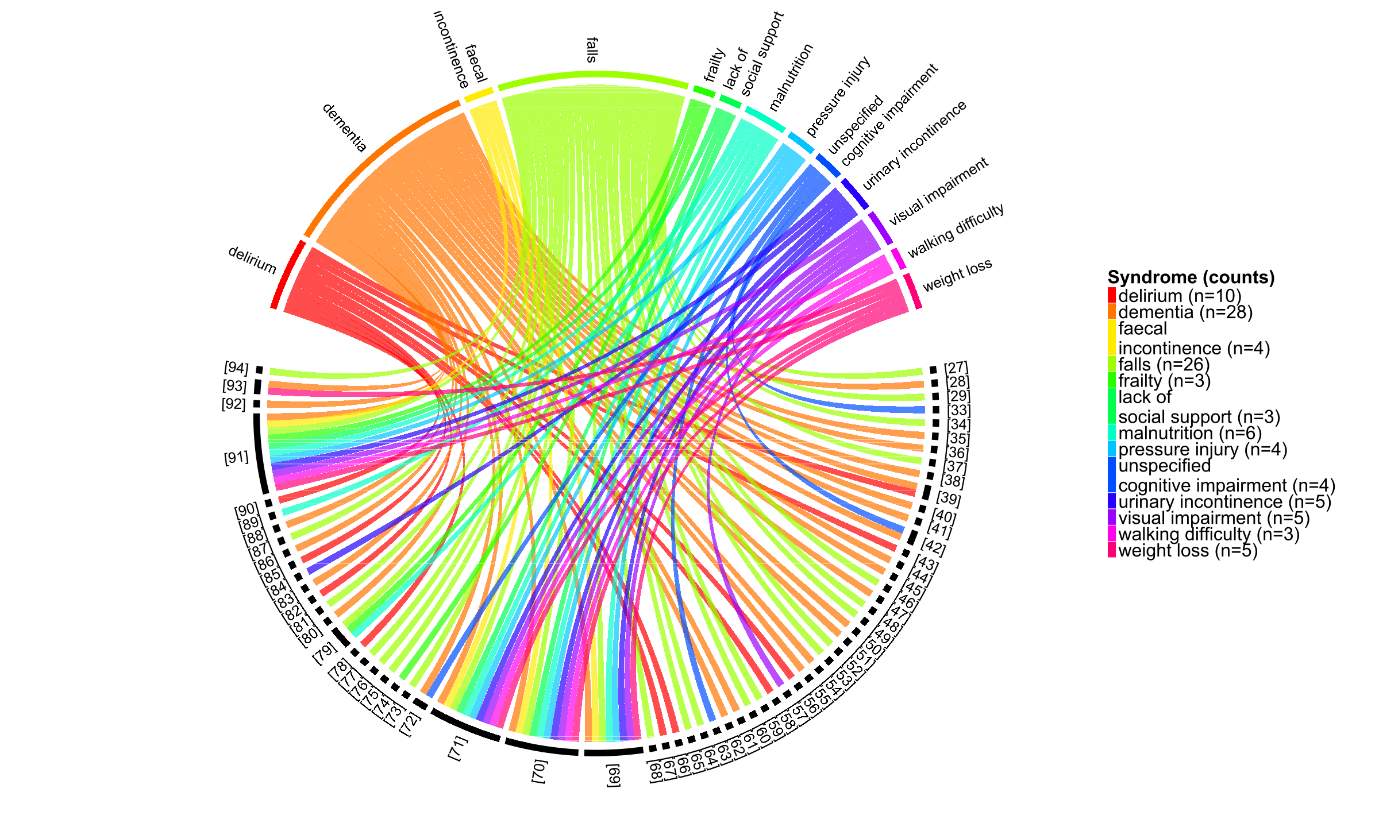


Figure 1 Chord diagram of authors (black sectors) and the geriatric syndromes they studied (coloured sectors). Sample size in the legend corresponds to the number of studies for the syndromes overall (not unique).


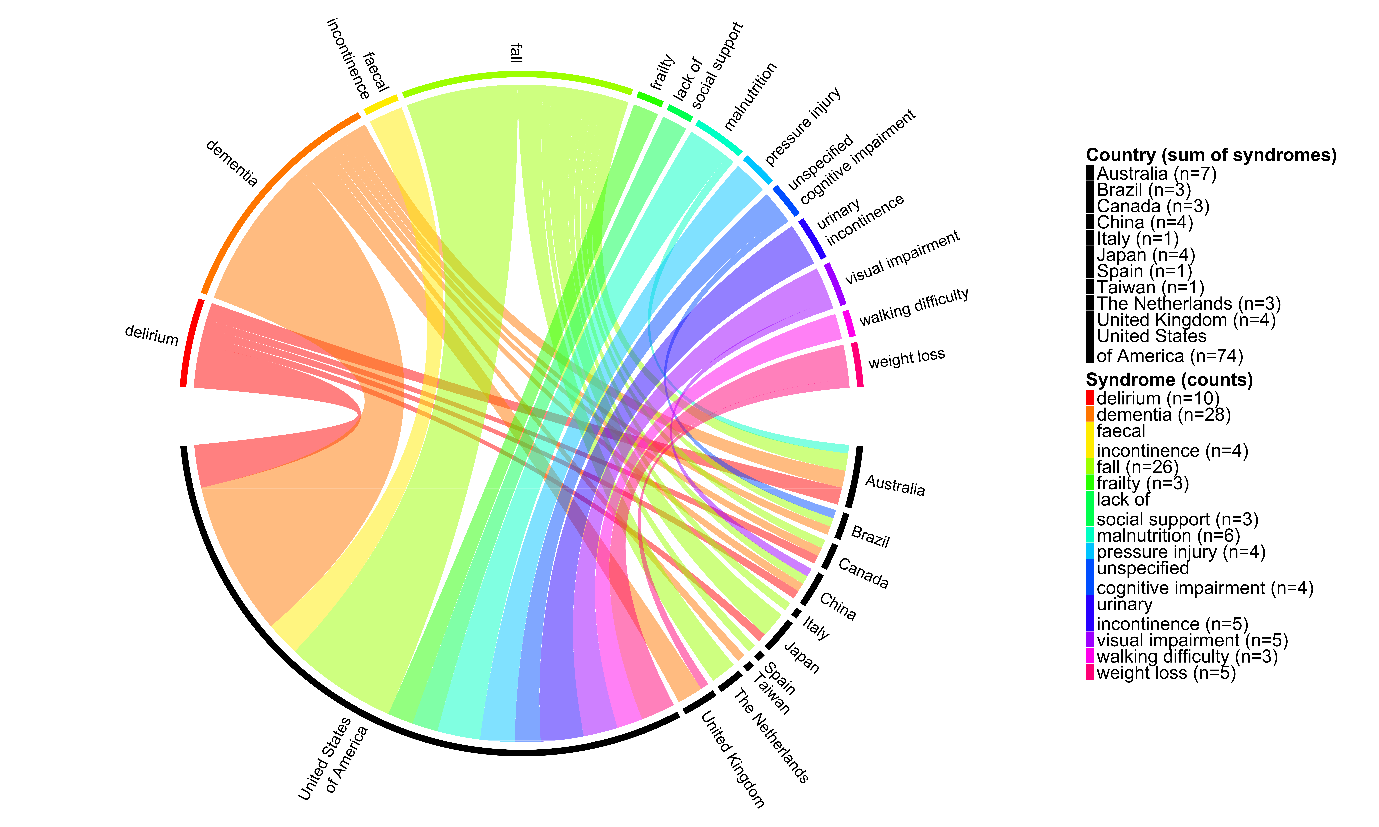


Figure 2 Chord diagram of countries (black sectors) and the sum of geriatric syndromes they studied (coloured sectors). Sample sizes in the legend correspond to the sum of countries and syndromes overall (not unique).

**Study references**

[27] Santos, H.D.P., Silva, A.P., Maciel, M.C.O., Burin, H.M.V., Urbanetto, J.S., Vieira, R.: Fall detection in EHR using word embeddings and deep learning. In: 2019 IEEE 19th International Conference on Bioinformatics and Bioengineering (BIBE), pp. 265–268 (2019). <https://doi.org/10.1109/BIBE.2019.00054>

[28] Panahi, S., Mayo, J., Kennedy, E., Christensen, L., Kamineni, S., Sagiraju, H.K.R., Cooper, T., Tate, D.F., Rupper, R., Pugh, M.J.: Identifying clinical phenotypes of frontotemporal dementia in post-9/11 era veterans using natural language processing. Frontiers in Neurology 15 (2024) <https://doi.org/10.3389/fneur.2024.1270688>

[29] Lorenzoni, G., Rampazzo, R., Buratin, A., Berchialla, P., Gregori, D.: Does the integration of pre-coded information with narratives improve in-hospital falls’ surveillance? Applied Sciences 11(10) (2021) <https://doi.org/10.3390/app11104406>

[33] Du, X., Novoa-Laurentiev, J., Plasek, J.M., Chuang, Y.-W., Wang, L., Marshall, G.A., Mueller, S.K., Chang, F., Datta, S., Paek, H., Lin, B., Wei, Q., Wang, X., Wang, J., Ding, H., Manion, F.J., Du, J., Bates, D.W., Zhou, L.: Enhancing early detection of cognitive decline in the elderly: a comparative study utilizing large language models in clinical notes. eBioMedicine 109 (2024) <https://doi.org/10.1016/j.ebiom.2024.105401>

[34] Cheligeer, C., Wu, G., Lee, S., Pan, J., Southern, D.A., Martin, E.A., Sapiro, N., Eastwood, C.A., Quan, H., Xu, Y.: Bert-based neural network for inpatient fall detection from electronic medical records: Retrospective cohort study. JMIR Medical Informatics 12 (2024) <https://doi.org/10.2196/48995>

[35] Laurentiev, J., Kim, D.H., Mahesri, M., Wang, K.-Y., Bessette, L.G., York, C., Zakoul, H., Lee, S.B., Zhou, L., Lin, K.J.: Identifying functional status impairment in people living with dementia through natural language processing of clinical documents: Cross-sectional study. Journal of Medical Internet Research 26, 47739 (2024) <https://doi.org/10.2196/47739>

[36] Sivarajkumar, S., Tam, T.Y.C., Mohammad, H.A., Viggiano, S., Oniani, D., Visweswaran, S., Wang, Y.: Extraction of sleep information from clinical notes of alzheimer’s disease patients using natural language processing. Journal of the American Medical Informatics Association 31(10), 2217–2227 (2024) <https://doi.org/10.1093/jamia/ocae177>

[37] Zhang, Y., Zhao, G., Zhao, Z., Luo, J., Feng, P., Tong, Y., Zhang, J., Tan, L., Sui, W.: Quantitative analysis of the causes of falls in adult hospitalized patients based on the perspective of text mining. Journal of Investigative Surgery 37(1), 2397578 (2024) <https://doi.org/10.1080/08941939.2024.2397578>

[38] Wu, W., Holkeboer, K.J., Kolawole, T.O., Carbone, L., Mahmoudi, E.: Natural language processing to identify social determinants of health in Alzheimer's disease and related dementia from electronic health records. Health Services Research 58(6), 1292–1302 (2023) <https://doi.org/10.1111/1475-6773.14210>

[39] Chen, L., Li, N., Zheng, Y., Gao, L., Ge, N., Xie, D., Yue, J.: A novel semi-automatic Chinese keywords instrument screening delirium based on electronic medical records. BMC Geriatrics 22(1), 779 (2022) <https://doi.org/10.1186/s12877-022-03474-w>

[40] Topaz, M., Adams, V., Wilson, P., Woo, K., Ryvicker, M.: Free-text documentation of dementia symptoms in home healthcare: A natural language processing study. Gerontology and Geriatric Medicine 6, 2333721420959861 (2020) <https://doi.org/10.1177/2333721420959861>

[41] Shao, Y., Todd, K., Shutes-David, A., Millard, S.P., Brown, K., Thomas, A., Chen, K., Wilson, K., Zeng, Q.T., Tsuang, D.W.: Identifying probable dementia in undiagnosed black and white Americans using machine learning in veterans health administration electronic health records. Big Data and Cognitive Computing 7(4) (2023) <https://doi.org/10.3390/bdcc7040167>

[42] Noori, A., Magdamo, C., Liu, X., Tyagi, T., Li, Z., Kondepudi, A., Alabsi, H., Rudmann, E., Wilcox, D., Brenner, L., Robbins, G.K., Moura, L., Zafar, S., Benson, N.M., Hsu, J., R Dickson, J., Serrano-Pozo, A., Hyman, B.T., Blacker, D., Westover, M.B., Mukerji, S.S., Das, S.: Development and evaluation of a natural language processing annotation tool to facilitate phenotyping of cognitive status in electronic health records: Diagnostic study. Journal of Medical Internet Research 24(8), 40384 (2022) <https://doi.org/10.2196/40384>

[43] Fu, S., Lopes, G.S., Pagali, S.R., Thorsteinsdottir, B., LeBrasseur, N.K., Wen, A., Liu, H., Rocca, W.A., Olson, J.E., St. Sauver, J., Sohn, S.: Ascertainment of delirium status using natural language processing from electronic health records. The Journals of Gerontology: Series A 77(3), 524–530 (2020) <https://doi.org/10.1093/gerona/glaa275>

[44] Li, S., Dexter, P., Ben-Miled, Z., Boustani, M.: Dementia risk prediction using decision-focused content selection from medical notes. Computers in Biology and Medicine 182, 109144 (2024) <https://doi.org/10.1016/j.compbiomed.2024.109144>

[45] Tsai, H., Yang, T.-W., Ou, K.-H., Su, T.-H., Lin, C., Chou, C.-F.: Multimodal attention network for dementia prediction. IEEE Journal of Biomedical and Health Informatics 28(11), 6918–6930 (2024) <https://doi.org/10.1109/JBHI.2024.3438885>

[46] Dormosh, N., Schut, M.C., Heymans, M.W., Maarsingh, O., Bouman, J., Velde, N., Abu-Hanna, A.: Predicting future falls in older people using natural language processing of general practitioners’ clinical notes. Age and Ageing 52(4), 046 (2023) <https://doi.org/10.1093/ageing/afad046>

[47] Zolnoori, M., Barr´on, Y., Song, J., Noble, J., Burgdorf, J., Ryvicker, M., Topaz, M.: HomeADScreen: Developing Alzheimer’s disease and related dementia risk identification model in home healthcare. International Journal of Medical Informatics 177, 105146 (2023) <https://doi.org/10.1016/j.ijmedinf.2023.105146>

[48] Mishra, A.K., Chappell, M.J., Emerson, S., Skubic, M.: Fall risk prediction in older adults using free-text nursing notes and medications in electronic health records. In: 2023 45th Annual International Conference of the IEEE Engineering in Medicine & Biology Society (EMBC), pp. 1–4 (2023). <https://doi.org/10.1109/EMBC40787.2023.10341127>

[49] Kawazoe, Y., Shimamoto, K., Shibata, D., Shinohara, E., Kawaguchi, H., Yamamoto, T.: Impact of a clinical text–based fall prediction model on preventing extended hospital stays for elderly inpatients: Model development and performance evaluation. JMIR Medical Informatics 10(7) (2022) <https://doi.org/10.2196/37913>

[50] Hane, C.A., Nori, V.S., Crown, W.H., Sanghavi, D.M., Bleicher, P.: Predicting onset of dementia using clinical notes and machine learning: Case-control study. JMIR Medical Informatics 8(6) (2020) <https://doi.org/10.2196/17819>

[51] Knapp, M., Chua, K.-C., Broadbent, M., Chang, C.-K., Fernandez, J.-L., Milea, D., Romeo, R., Lovestone, S., Spencer, M., Thompson, G., Stewart, R., Hayes, R.D.: Predictors of care home and hospital admissions and their costs for older people with Alzheimer’s disease: findings from a large london case register. BMJ Open 6(11) (2016) <https://doi.org/10.1136/bmjopen-2016-013591>

[52] Topaz, M., Murga, L., Gaddis, K.M., McDonald, M.V., Bar-Bachar, O., Goldberg, Y., Bowles, K.H.: Mining fall-related information in clinical notes: Comparison of rule-based and novel word embedding-based machine learning approaches. Journal of Biomedical Informatics 90, 103103 (2019) <https://doi.org/10.1016/j.jbi.2019.103103>

[53] Ge, W., Godeiro Coelho, L.M., Donahue, M.A., Rice, H.J., Blacker, D., Hsu, J., Newhouse, J.P., Hernandez-Diaz, S., Haneuse, S., Westover, B., Moura, L.M.V.R.: Automated identification of fall-related injuries in unstructured clinical notes. American Journal of Epidemiology, 240 (2024) <https://doi.org/10.1093/aje/kwae240>

[54] Vithanage, D., Zhu, Y., Zhang, Z., Deng, C., Yin, M., Yu, P.: Extracting symptoms of agitation in dementia from free-text nursing notes using advanced natural language processing. Studies in Health Technology and Informatics 310, 700–704 (2024) <https://doi.org/10.3233/SHTI231055>

[55] Prakash, R., Dupre, M.E., Østbye, T., Xu, H.: Extracting critical information from unstructured clinicians’ notes data to identify dementia severity using a rule-based approach: Feasibility study. JMIR Aging 7, 57926 (2024) <https://doi.org/10.2196/57926>

[56] Amjad, S., Holmes, N.E., Kishore, K., Young, M., Bailey, J., Bellomo, R., Verspoor, K.: Advancing delirium classification: A clinical notes-based natural language processing-supported machine learning model. Intelligence-Based Medicine 9, 100140 (2024) <https://doi.org/10.1016/j.ibmed.2024.100140>

[57] Guo, Y., Huang, C., Sheng, Y., Zhang, W., Ye, X., Lian, H., Xu, J., Chen, Y.: Improve the efficiency and accuracy of ophthalmologists’ clinical decision-making based on AI technology. BMC Medical Informatics and Decision Making 24(1), 192 (2024) <https://doi.org/10.1186/s12911-024-02587-z>

[58] St. Sauver, J., Fu, S., Sohn, S., Weston, S., Fan, C., Olson, J., Thorsteinsdottir, B., LeBrasseur, N., Pagali, S., Rocca, W., Liu, H.: Identification of delirium from real-world electronic health record clinical notes. Journal of Clinical and Translational Science 7(1) (2023) <https://doi.org/10.1017/cts.2023.610>

[59] Millet, A., Madrid, A., Alonso-Weber, J.M., Rodrıguez-Manas, L., Perez-Rodra-Guez, R.: Machine learning techniques applied to the development of a fall risk index for older adults. IEEE Access 11, 84795–84809 (2023) <https://doi.org/10.1109/ACCESS.2023.3299489>

[60] Powell, J.M., Guo, Y., Sarker, A., McKay, J.L.: Classification of fall types in parkinson’s disease from self-report data using natural language processing. In: Juarez, J.M., Marcos, M., Stiglic, G., Tucker, A. (eds.) Artificial Intelligence in Medicine, pp. 163–172. Springer, Cham (2023). <https://doi.org/10.1007/978-3-031-34344-5_20>

[61] Maclagan, L.C., Abdalla, M., Harris, D.A., Stukel, T.A., Chen, B., Candido, E., Swartz, R.H., Iaboni, A., Jaakkimainen, R.L., Bronskill, S.E.: Can patients with dementia be identified in primary care electronic medical records using natural language processing? Journal of Healthcare Informatics Research 7(1), 42–58 (2023) <https://doi.org/10.1007/s41666-023-00125-6>

[62] Liu, M., Beare, R., Collyer, T., Andrew, N., Srikanth, V.: Leveraging natural language processing and clinical notes for dementia detection. In: Naumann, T., Ben Abacha, A., Bethard, S., Roberts, K., Rumshisky, A. (eds.) Proceedings of the 5th Clinical Natural Language Processing Workshop, pp. 150–155. Association for Computational Linguistics, Toronto, Canada (2023). <https://doi.org/10.18653/v1/2023.clinicalnlp-1.20>

[63] Penfold, R.B., Carrell, D.S., Cronkite, D.J., Pabiniak, C., Dodd, T., Glass, A.M., Johnson, E., Thompson, E., Arrighi, H.M., Stang, P.E.: Development of a machine learning model to predict mild cognitive impairment using natural language processing in the absence of screening. BMC Medical Informatics and Decision Making 22(1), 129 (2022) <https://doi.org/10.1186/s12911-022-01864-z>

[64] Tohira, H., Finn, J., Ball, S., Brink, D., Buzzacott, P.: Machine learning and natural language processing to identify falls in electronic patient care records from ambulance attendances. Informatics for Health and Social Care 47(4), 403–413 (2022) <https://doi.org/10.1080/17538157.2021.2019038>

[65] Fu, S., Thorsteinsdottir, B., Zhang, X., Lopes, G.S., Pagali, S.R., LeBrasseur, N.K., Wen, A., Liu, H., Rocca, W.A., Olson, J.E., Sauver, J.S., Sohn, S.: A hybrid model to identify fall occurrence from electronic health records. International Journal of Medical Informatics 162, 104736 (2022) <https://doi.org/10.1016/j.ijmedinf.2022.104736>

[66] Wang, L., Zhang, Y., Chignell, M., Shan, B., Sheehan, K.A., Razak, F., Verma, A.: Boosting delirium identification accuracy with sentiment-based natural language processing: Mixed methods study. JMIR Medical Informatics 10(12), 38161 (2022) <https://doi.org/10.2196/38161>

[67] Ge, W., Alabsi, H., Jain, A., Ye, E., Sun, H., Fernandes, M., Magdamo, C., Tesh, R.A., Collens, S.I., Newhouse, A., MVR Moura, L., Zafar, S., Hsu, J., Akeju, O., Robbins, G.K., Mukerji, S.S., Das, S., Westover, M.B.: Identifying patients with delirium based on unstructured clinical notes: Observational study. JMIR Formative Research 6(6), 33834 (2022) <https://doi.org/10.2196/33834>

[68] Nakatani, H., Nakao, M., Uchiyama, H., Toyoshiba, H., Ochiai, C.: Predicting inpatient falls using natural language processing of nursing records obtained from Japanese electronic medical records: Case-control study. JMIR Medical Informatics 8(4) (2020) <https://doi.org/10.2196/16970>

[69] Kharrazi, H., Anzaldi, L.J., Hernandez, L., Davison, A., Boyd, C.M., Leff, B., Kimura, J., Weiner, J.P.: The value of unstructured electronic health record data in geriatric syndrome case identification. Journal of the American Geriatrics Society 66(8), 1499–1507 (2018) <https://doi.org/10.1111/jgs.15411>

[70] Chen, T., Dredze, M., Weiner, J.P., Hernandez, L., Kimura, J., Kharrazi, H.: Extraction of geriatric syndromes from electronic health record clinical notes: Assessment of statistical natural language processing methods. JMIR Medical Informatics 7(1), 13039 (2019) <https://doi.org/10.2196/13039>

[71] Chen, T., Dredze, M., Weiner, J.P., Kharrazi, H.: Identifying vulnerable older adult populations by contextualizing geriatric syndrome information in clinical notes of electronic health records. Journal of the American Medical Informatics Association 26(8-9), 787–795 (2019) <https://doi.org/10.1093/jamia/ocz093>

[72] Moreira, L.B., Namen, A.A.: A hybrid data mining model for diagnosis of patients with clinical suspicion of dementia. Computer Methods and Programs in Biomedicine 165, 139–149 (2018) <https://doi.org/10.1016/j.cmpb.2018.08.016>

[73] Patterson, B.W., Jacobsohn, G.C., Shah, M.N., Song, Y., Maru, A., Venkatesh, A.K., Zhong, M., Taylor, K., Hamedani, A.G., Mendon¸ca, E.A.: Development and validation of a pragmatic natural language processing approach to identifying falls in older adults in the emergency department. BMC Medical Informatics and Decision Making 19(1), 138 (2019) <https://doi.org/10.1186/s12911-019-0843-7>

[74] Zeng-Treitler, Q., Shao, Y., Cheng, Y., Doing-Harris, K., Shah, R.U., Weir, C.R., Bray, B.E.: Extracting frailty status for post surgical mortality prediction. In: IADIS International Conference e-Health 2018 (2018). <https://www.iadisportal.org/digital-library/extracting-frailty-status-for-post-surgical-mortality-prediction>

[75] Toyabe, S.-I.: Detecting inpatient falls by using natural language processing of electronic medical records. BMC Health Services Research 12(1), 448 (2012) <https://doi.org/10.1186/1472-6963-12-448>

[76] Tremblay, M.C., Berndt, D.J., Foulis, P., Luther, S.L.: Utilizing text mining techniques to identify fall related injuries. In: Americas Conference on Information Systems (2005). <https://aisel.aisnet.org/amcis2005/109>

[77] Tremblay, M.C., Berndt, D.J., Luther, S.L., Foulis, P.R., French, D.D.: Identifying fall-related injuries: Text mining the electronic medical record. Information Technology and Management 10(4), 253–265 (2009) <https://doi.org/10.1007/s10799-009-0061-6>

[78] Pagali, S.R., Kumar, R., Fu, S., Sohn, S., Yousufuddin, M.: Natural language processing CAM algorithm improves delirium detection compared with conventional methods. American Journal of Medical Quality 38(1), 17–22 (2023) <https://doi.org/10.1097/jmq.0000000000000090>

[79] Martin, J.A., Crane-Droesch, A., Lapite, F.C., Puhl, J.C., Kmiec, T.E., Silvestri, J.A., Ungar, L.H., Kinosian, B.P., Himes, B.E., Hubbard, R.A., Diamond, J.M., Ahya, V., Sims, M.W., Halpern, S.D., Weissman, G.E.: Development and validation of a prediction model for actionable aspects of frailty in the text of clinicians’ encounter notes. Journal of the American Medical Informatics Association 29(1), 109–119 (2021) <https://doi.org/10.1093/jamia/ocab248>

[80] Gibson, L.L., Mueller, C., Stewart, R., Aarsland, D.: Characteristics associated with progression to probable dementia with lewy bodies in a cohort with very late-onset psychosis. Psychological Medicine 54(12), 1–10 (2024) <https://doi.org/10.1017/s0033291724001922>

[81] Dormosh, N., Abu-Hanna, A., Calixto, I., Schut, M.C., Heymans, M.W., Velde, N.: Topic evolution before fall incidents in new fallers through natural language processing of general practitioners’ clinical notes. Age and Ageing 53(2), 016 (2024) <https://doi.org/10.1093/ageing/afae016>

[82] Chen, A., Paredes, D., Yu, Z., Lou, X., Brunson, R., Thomas, J.N., Martinez, K.A., Lucero, R.J., Magoc, T., Solberg, L.M., Snigurska, U.A., Ser, S.E., Prosperi, M., Bian, J., Bjarnadottir, R.I., Wu, Y.: Identifying symptoms of delirium from clinical narratives using natural language processing. In: 2024 IEEE 12th International Conference on Healthcare Informatics (ICHI), pp. 305–311 (2024). <https://doi.org/10.1109/ICHI61247.2024.00046>

[83] Ryvicker, M., Barron, Y., Song, J., Zolnoori, M., Shah, S., Burgdorf, J.G., Noble, J.M., Topaz, M.: Using natural language processing to identify home health care patients at risk for diagnosis of Alzheimer’s disease and related dementias. Journal of Applied Gerontology 43(10), 1461–1472 (2024) <https://doi.org/10.1177/07334648241242321>

[84] Scharp, D., Song, J., Hobensack, M., Palmer, M.H., Barcelona, V., Topaz, M.: Applying natural language processing to understand symptoms among older adult home healthcare patients with urinary incontinence. Journal of Nursing Scholarship 57(1), 152–164 (2025) <https://doi.org/10.1111/jnu.13038>

[85] Miyazawa, Y., Katsuta, N., Nara, T., Nojiri, S., Naito, T., Hiki, M., Ichikawa, M., Takeshita, Y., Kato, T., Okumura, M., Tobita, M.: Identification of risk factors for the onset of delirium associated with covid-19 by mining nursing records. PLOS ONE 19(1), 1–14 (2024) <https://doi.org/10.1371/journal.pone.0296760>

[86] Oh, I.Y., Schindler, S.E., Ghoshal, N., Lai, A.M., Payne, P.R.O., Gupta, A.: Extraction of clinical phenotypes for alzheimer’s disease dementia from clinical notes using natural language processing. Journal of the American Medical Informatics Association Open 6(1), 014 (2023) <https://doi.org/10.1093/jamiaopen/ooad014>

[87] Altuhaifa, F., Al Tuhaifa, D., Al Ribh, E., Al Rebh, E.: Identifying and defining entities associated with fall risk factors events found in fall risk assessment tools. Computer Methods and Programs in Biomedicine Update 3, 100105 (2023) <https://doi.org/10.1016/j.cmpbup.2023.100105>

[88] Chen, Z., Zhang, H., Yang, X., Wu, S., He, X., Xu, J., Guo, J., Prosperi, M., Wang, F., Xu, H., Chen, Y., Hu, H., DeKosky, S.T., Farrer, M., Guo, Y., Wu, Y., Bian, J.: Assess the documentation of cognitive tests and biomarkers in electronic health records via natural language processing for Alzheimer’s disease and related dementias. International Journal of Medical Informatics 170, 104973 (2023) <https://doi.org/10.1016/j.ijmedinf.2022.104973>

[89] Alkhalaf, M., Zhang, Z., Chang, H.-C.R., Wei, W., Yin, M., Deng, C., Yu, P.: Malnutrition and its contributing factors for older people living in residential aged care facilities: Insights from natural language processing of aged care records. Technology and Health Care 31(6), 2267–2278 (2023) <https://doi.org/10.3233/thc-230229>

[90] Young, M., Holmes, N., Robbins, R., Marhoon, N., Amjad, S., Neto, A.S., Bellomo, R.: Natural language processing to assess the epidemiology of delirium-suggestive behavioural disturbances in critically ill patients. Critical Care and Resuscitation 23(2), 144–153 (2021) <https://doi.org/10.51893/2021.2.oa1>

[91] Anzaldi, L.J., Davison, A., Boyd, C.M., Leff, B., Kharrazi, H.: Comparing clinician descriptions of frailty and geriatric syndromes using electronic health records: a retrospective cohort study. BMC Geriatrics 17(1) (2017) <https://doi.org/10.1186/s12877-017-0645-7>

[92] Zhou, X., Wang, Y., Sohn, S., Therneau, T.M., Liu, H., Knopman, D.S.: Automatic extraction and assessment of lifestyle exposures for Alzheimer’s disease using natural language processing. International Journal of Medical Informatics 130, 103943 (2019) <https://doi.org/10.1016/j.ijmedinf.2019.08.003>

[93] Soysal, P., Tan, S.G., Rogowska, M., Jawad, S., Smith, L., Veronese, N., Tsiptsios, D., Tsamakis, K., Stewart, R., Mueller, C.: Weight loss in Alzheimer’s disease, vascular dementia and dementia with lewy bodies: Impact on mortality and hospitalization by dementia subtype. International Journal of Geriatric Psychiatry 37(2) (2022) <https://doi.org/10.1002/gps.5659>

[94] Leurs, W.L.M., Lammers, L.A.S., Compagner, W.N., Groeneveld, M., Korsten, E.H.H.M., van der Linden, C.M.J.: Text mining in nursing notes for text characteristics associated with in-hospital falls in older adults: A case-control pilot study. Aging and Health Research 2(2), 100078 (2022) <https://doi.org/10.1016/j.ahr.2022.100078>
